# Global Navigation Process Simulation Based on Different Types of Gravity Data

**DOI:** 10.3390/s20205859

**Published:** 2020-10-16

**Authors:** Ivan Lytkin, Oleg Tarasov

**Affiliations:** Synest LLC, Ulica Malygina 5, Tyumen 625048, Russia; nata555li@mail.ru

**Keywords:** gravity-aided navigation, gravity anomaly, modeling, numerical simulation

## Abstract

A theoretical study on the feasibility of global navigation based on three different types of gravity data was performed. A computer simulation of gravity-aided navigation was performed for three models of sections of the Earth’s surface with gravity anomalies distributed as specified. For navigation, three types of data sources were used, e.g., the gravity vector magnitude, three orthogonal projections of the gravity vector, and five independent components of the full gravity tensor. For each data source, when searching a specified route, the dependencies of the number of the identified true and false points were determined in accordance with the measurement error specified. The problem of determining the true route on the set of the identified points is briefly reviewed. General conclusions are presented regarding the practical applicability of the reviewed data sources to the problem of global navigation.

## 1. Introduction

Global navigation systems are an important element for the functioning of the majority of modern transport systems, from civilian vehicles and nuclear submarines to autonomous drones. Delivery of cargo by water and air, passenger transport, and space missions are barely conceivable without location tools, without the use of satellite and radar systems, or without hybrid solutions that combine various tools into a single navigation system. However, any navigation system has its limitations and operational features.

For example, satellite navigation tools, even taking into account open access to satellite networks and high positioning accuracy, fail to function in polar regions and during strong magnetic storms [1]. In addition, at any time and in any territory, the system owners can disable them, and satellite signal scan can be suppressed or distorted [2]. If satellite systems are unavailable, ground navigation aids can be used for global navigation. However, ground-based systems are characterized by limited coverage and insufficient accuracy and depend on the state of the ionosphere [3]. One promising direction for the creation of alternative systems for independent global navigation is the use of the Earth’s gravity field [4,5,6].

A feature of a global navigation system based on tracking according to gravity field data is its independence from external auxiliary systems and its immunity from interference and distortion of a useful signal. A topographic map can be used for navigation with the characteristics of the gravity field referenced. Requirements for the referenced point density, total area of the map, and the period of relevance of the information presented depend on the characteristics of the gravity field selected for navigation.

The aim of this study was theoretical research on the problems of gravity navigation based on various types of gravity data. Using an open database of gravity anomalies, we have built three virtual models of the Earth’s surface, for which we calculated values sets of three types of initial data and developed a simple navigation algorithm to demonstrate the general and specific problems of gravitational navigation.

To simulate the process of global gravity-aided navigation, we have defined three sets of characteristics [7,8] that represent the state of the Earth’s gravity field at a referenced point: (1) the gravity vector magnitude $\left|\bar{g}\right|$; (2) three projections of the acceleration vector on the orthogonal axes $g_{x}$, $g_{y}$, $g_{z}$; and (3) five independent components of the gravity tensor $T_{xx}$, $T_{xy}$, $T_{xz}$, $T_{yy}$, $T_{yz}$ [9]. For all datasets, the maximum permissible errors in the measurements of the Earth’s gravity field have been defined where the method of navigation gives the correct result (that is, among the potential solutions found, the desired route can be guaranteed).

The navigation problem was simulated in the author’s computer simulation environment for three variants of the distribution of the gravity field, arbitrarily divided by the level of the gravity gradient from relatively low to high. Publicly available data from the Gravity Field and Steady-State Ocean Circulation Explorer (GOCE) satellite mission (2009–2013) [10] provided the realistic prototypes for the selected distributions. On the basis of the information generated from satellite gravimetric surveys, digital copies of these regions have been formed, consisting of virtual geological objects specifically located and mass which, in aggregate, reflect the gravity effect, and generally correspond to the prototypes.

The computer simulation was based on an algorithm designed for searching a fixed-length route among points on a base map, with the referenced values of gravity components of the gravity field tensor. The algorithm resulted in a set of potential solutions to the problem of navigation in the form of points comprising the desired route. The number and characteristics of the distribution of the obtained solutions varied with the simulation environments. The characteristics of the route built to demonstrate the main problems of gravitational navigation (such as route length and the number of points) are determined as a result of the series of computer experiments to show the features in the accumulation of basic errors while using the selected type of gravity data. In addition, in order to simplify the basic navigation algorithm, the actual distance between points of the route was added into the basic simulation conditions (in practice, this information can be received using Inertial Navigation Systems (INS)).

According to the simulation results, a general analysis of the features of application of various characteristics of the gravity field for navigation was conducted.

It should also be noted that the task of this article was to overview the immediately possible approaches to the implementation of gravity navigation without taking into account the nuances of technical implementation of navigation devices on the principles described. For that reason, we deliberately “idealize” conditions for navigation signal acquisition and do not consider the possibility of integration of gravity navigation in existing navigation systems in detail. This material is planned as a topic for further research by the authors.

## 2. Simulation

To serve as virtual polygons, three-dimensional plots of the Earth’s interior (200 × 200 × 40 km^2^) simulated and divided by the nature of the gravity anomalies that was based on data from satellite gravimetric surveys (Table 1):

(1) A smooth field with uniformly distributed gravity heterogeneities, characteristic of the Nadym lowland, located on the immature West Siberian platform.

(2) A field with distinct local heterogeneities and gravity gradients corresponding to the transition from the Nadym lowland to the Poluy highland (zone of the Poluy anticlinorium).

(3) A field with contrasting anomalies of the folded belt (the Mugodzhary Mountains, the southern spur of the Ural Mountains, Kazakhstan).

For the sake of brevity, the regions defined will be referred to as regions with low- (gradients on the order of 0.1 mGal/km), medium- (gradients of 1 mGal/km), and high-amplitude (gradients of 10 mGal/km) anomalies, respectively.

For the calculation of gravity anomalies, we used n = 21 mass points $m_{i}$ in the range of 1⋅10^12^–2⋅10^16^ kg, distributed over an area of 200 × 200 km^2^ at depths of 12–40 km. Coordinates of the mass points are denoted by $\left(x_{i,\;}y_{i,\;}z_{i}\right)$. The grid spacing was 8 km for all model regions.

Data arrays for the three characteristics of the gravity field were calculated:

(1) gravity vector magnitude, $\left|\bar{g}\right|$.

(2) three projections of $\bar{g}$ onto the corresponding Cartesian axes, $g_{x}$, $g_{y}$, $g_{z}$.

(3) five independent components of the full gravity tensor, $T_{xx}$, $T_{xy}$, $T_{xz}$, $T_{yy}$, $T_{yz}$.

For the calculation of the components of the full gravity tensor, the calculated distance between the arms of the virtual gravity gradiometer was 5 m.

For all regions specified, three components of the gravity acceleration vector from this set of mass points, this vector amplitude, and 5 independent components of the full gravity tensor were calculated at a height of z=0 km.

(1) $$g_{x}=\;\sum_{i=1}^{n}\frac{Gm_{i}\Delta x_{i}}{r_{i}^{3}}$$

(2) $$g_{y}=\;\sum_{i=1}^{n}\frac{Gm_{i}\Delta y_{i}}{r_{i}^{3}}$$

(3) $$g_{z}=\;\sum_{i=1}^{n}\frac{Gm_{i}\Delta z_{i}}{r_{i}^{3}}$$

(4) $$\left|g\right|=\;\sqrt{g_{x}^{2}+g_{y}^{2}+g_{z}^{2}}$$

(5) $$T_{xx}=\sum_{i=1}^{n}\frac{Gm_{i}}{r_{i}^{5}}\left(-3{\left(x-x_{i}\right)}^{2}+r_{i}^{2}\right)$$

(6) $$T_{xy}=\sum_{i=1}^{n}\frac{Gm_{i}}{r_{i}^{5}}\left(-3\left(x-x_{i}\right)\left(y-y_{i}\right)\right)$$

(7) $$T_{xz}=\sum_{i=1}^{n}\frac{Gm_{i}}{r_{i}^{5}}\left(-3\left(x-x_{i}\right)\left(z-z_{i}\right)\right)$$

(8) $$T_{yy}=\sum_{i=1}^{n}\frac{Gm_{i}}{r_{i}^{5}}\left(-3{\left(y-y_{i}\right)}^{2}+r_{i}^{2}\right)$$

(9) $$T_{yz}=\sum_{i=1}^{n}\frac{Gm_{i}}{r_{i}^{5}}\left(-3\left(y-y_{i}\right)\left(z-z_{i}\right)\right)$$

where

G = 6.67408⋅10^−11^ m^3^⋅kg^−1^⋅s^−2^, the gravitational constant [11],
$\Delta x_{i}=x-x_{i}$, $\Delta y_{i}=y-y_{i}$, $\Delta z_{i}=z-z_{i}$, the Cartesian projections of the distances between the grid node and the *i*-th mass,
$r_{i}^{2}=\Delta x_{i}^{2}+\Delta y_{i}^{2}+\Delta z_{i}^{2}$, the distance between a grid node and the *i*-th mass.

**Figure 1:**
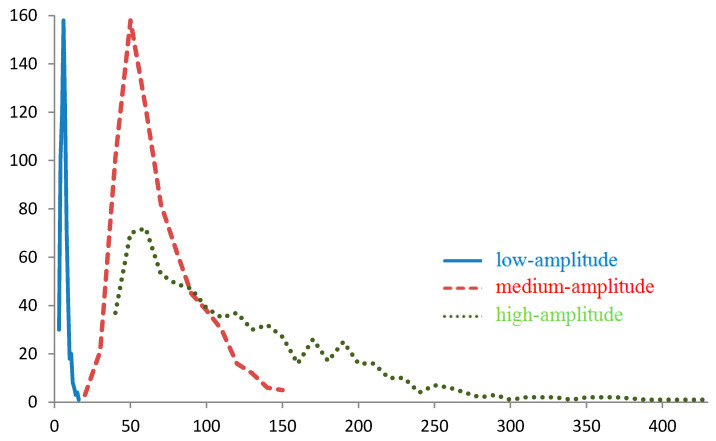
Distribution of points by $\left|\bar{g}\right|$ for regions with low-amplitude, medium-amplitude, and high-amplitude gravity anomalies, mGal.

**Figure 2:**
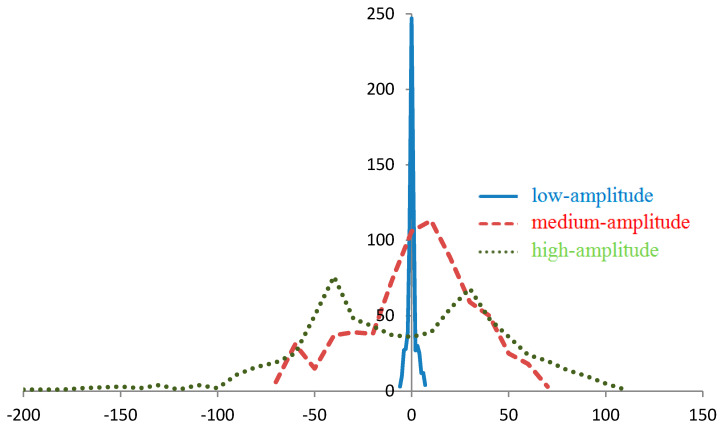
Distribution of points by $g_{x}$ for regions with low-amplitude, medium-amplitude, and high-amplitude gravity anomalies, mGal.

**Figure 3:**
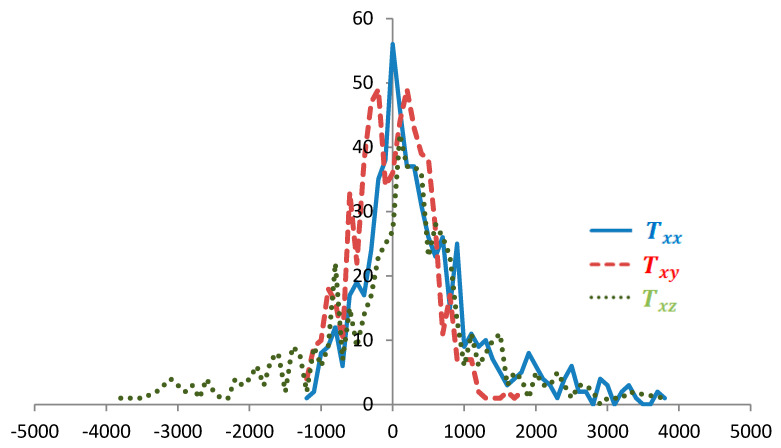
Distribution of points by $T_{xx}$, $T_{xy}$, $T_{xz}$ for the region with medium-amplitude gravity anomalies, E.

We consider the features of the distribution of these characteristics for the calculated regions (Figure 1, Figure 2 and Figure 3). Distributions for $g_{y}$, $g_{z}$, $T_{yy}$, and $T_{yz}$ are not provided because they are close (considering the opposing signs) to the distributions with respect to $g_{x}$, $\left|\bar{g}\right|$, $T_{xx}$ and $T_{xz}$, respectively.

The nature of the distributions of values $\left|\bar{g}\right|$ and of projections $\bar{g}$ changes with the magnitude of the anomalies for the region under study: the greater the anomalies are, the more asymmetric and non-monotonic the distributions become (Figure 1), which can be explained by the peculiarities of the mass distributions over various areas of the map. Thus, the distribution of the $g_{x}$ component for the foothills and the mountainous region actually reflects the shape of the anomalies on the map.

As the amplitude of the anomalies increases, variations in the values $\left|\bar{g}\right|$ and projections $\left|\bar{g}\right|$ also increase (Figure 1 and Figure 2), and the number of grid points corresponding to the specified interval of these values decreases. Therefore, with the same measurement error for regions with high-amplitude anomalies, on average, fewer points will be found that correspond to the value $\left|\bar{g}\right|$ or projection $\bar{g}$ specified.

For example, for the values of $g_{x}\approx 0$ for the region with low-amplitude anomalies, approximately 250 points can be distinguished, while for the regions with medium- and high-amplitude anomalies 100 and 35 points, respectively, can be distinguished (Figure 2). In the areas of local extreme points, a deviation from this rule can be observed. Therefore, for $g_{x}\approx -\;50\;$ mGal, the number of points in the region with high-amplitude anomalies (50 points) is greater than in the medium-amplitude anomalies region (15 points) due to the maximum of the first distribution and the minimum of the second distribution.

The distributions by tensor components have a more pronounced non-monotonic nature (Figure 3) than those by modules and by acceleration projections, i.e., the former is more sensitive to local anomalies. Thus, local maxima corresponding to the mass points specified in the model are clearly visible in the distributions by $T_{xx}$ and $T_{xz}$. In this case, the distributions of the same tensor components for regions with different anomaly amplitudes coincide qualitatively, with allowances being made for the amplitudes. In addition, the distributions of grid points by all tensor components are not shifted with respect to zero (like the distribution by $\left|\bar{g}\right|$ and $g_{z}$), and most of the points have a zero tensor value (unlike the distribution by $\left|\bar{g}\right|$ and components $\bar{g}$). This finding suggests that navigation equipment based on data from the components of the gravity tensor designed even for theregions with high-amplitude gravity anomalies would have small zero drift levels and high sensitivity.

The test navigation route included 7 points on a broken line running across the map from southwest to northeast. The height of the route points is taken as z=0 m. For the sake of simplicity, the points belong to the global grid nodes containing the calculated gravity characteristics for the entire area (which are the benchmark for all virtual measurements). Reference data also have been calculated for a height of z=0 m.

We denote the route points in the sequence order as d =0, d =1 … d =D. Table 2 shows the route points referenced to the coordinates of the grid with a spacing of 8 km on the area X∈ [−100,000;100,000], Y∈ [−100,000;100,000], Z= 0 m.

## 3. Method of Searching the Route Points

The basic condition for gravitational navigation is sufficient detailing of the gravitational anomalies captured on the ground. The navigation method should include the step of finding points with gravity characteristics close to those of the observer’s location points (route points) and the processing of the resulting data (screening the points, combining them into a route, and validating the route).

The values of the calculated gravity characteristics for the route points are shown in Table 3, Table 4 and Table 5.

We assign the following coordinates to the first point of the route: $x_{0}$= 0, $y_{0}$= 0, $z_{0}$= 0.

Assuming that in addition to the values of gravity, the distances between the route points are also known, we can calculate the coordinates of the following points of the route relative to the origin (points d=0 (0, 0, 0)).

Accordingly, we can define a route that does not refer to the global area as a polygonal line with D vertices ($x_{d}$, $y_{d}$).

Depending on the source data type selected for the navigation, for each route point d,  there are the following data arrays:

(1) the gravity acceleration vector magnitude, ${\left|\bar{g}\right|}_{d}$

(2) the gravity vector projections on the axis, $g_{x,d}$, $g_{y,d}$, $g_{z,d}$

(3) the five tensor components, $T_{xx,d}$, $T_{yy,d}$, $T_{xy,d}$, $T_{xz,d}$, $T_{yz,d}$

We then have a matrix of the route that is not referenced to the map, and the measured values of the gravity field characteristics are presented in Table 6.

We denote the known values of the gravity acceleration vector amplitude and the projections of the acceleration vector and the components of the gravity tensor at the vertices of the grid as $\left|\bar{g}\right|$, $g_{x}$, $g_{y}$, $g_{z}$ and $T_{xx}$, $T_{yy}$, $T_{xy}$, $T_{xz}$, $T_{yz}$.

Let ε be the standard deviation of measurements by a virtual instrument, designed as a criterion for referencing a measured point on a route to points of the base map.

For the d point of the route, several (or zero) points on the map are found. To distinguish these points, we introduce the sequence numbers, $j\in \left[1,\;J_{d}\right]$.

We denote the membership function of point d of the route for the region with the calculated values of the acceleration vector magnitude as $MFM_{d,j}$; for the region with the values of the projections of the acceleration vector as $MFP_{x,d,j}$, $MFP_{y,d,j}$, $MFP_{z,d,j}$; and for the region with the calculated values of the components of the tensor as $MFT_{xx,d,j}$, $MFT_{yy,d,j}$, $MFT_{xy,d,j}$, $MFT_{xz,d,j}$, $MFT_{yz,d,j}$.

Thus,

(10) $$MFM_{d,j}=\{\begin{array}{c} 1,\;if{\left(\left|\bar{g}\right|-{\left|\bar{g}\right|}_{d,j}\right)}^{2}\leq \varepsilon^{2}\\ 0,\;if{\left(\left|\bar{g}\right|-{\left|\bar{g}\right|}_{d,j}\right)}^{2}>\varepsilon^{2} \end{array}$$

where $\Delta {\left|\bar{g}\right|}_{d,j}=\sqrt{{\left(\left|\bar{g}\right|-{\left|\bar{g}\right|}_{d,j}\right)}^{2}}$−acceleration amplitude discrepancy.

For the projection of the acceleration vector, $g_{x}$, the membership function is

(11) $$MFP_{x,d,j}=\{\begin{array}{c} 1,\;if{\left(g_{x}-g_{x,d,j}\right)}^{2}\leq \varepsilon^{2}\\ 0,\;if{\left(g_{x}-g_{x,d,j}\right)}^{2}>\varepsilon^{2} \end{array}$$

where $\Delta g_{x,d,j}=\sqrt{{\left(g_{x}-g_{x,d,j}\right)}^{2}}$− acceleration vector projection discrepancy.

For the tensor component $T_{xx}$, the membership function is

(12) $$MFT_{xx,d,j}=\{\begin{array}{c} 1,\;if{\left(T_{xx}-T_{xx,d,j}\right)}^{2}\leq \varepsilon^{2}\\ 0,\;if{\left(T_{xx}-T_{xx,d,j}\right)}^{2}>\varepsilon^{2} \end{array}$$

where $\Delta T_{xx,d,j}=\sqrt{{\left(T_{xx}-T_{xx,d,j}\right)}^{2}}$−xx tensor component discrepancy.

Similarly, let us construct $MFP_{y,d,j}$, $MFP_{z,d,j}$ for the two other acceleration projections, $g_{y}$ and $g_{z}$, as well as $MFT_{yy,d,j}$, $MFT_{xy,d,j}$, $MFT_{xz,d,j}$, $MFT_{yz,d,j}$ for the remaining four tensor components, $T_{yy}$, $T_{xy}$, $T_{xz}$, $T_{yz}$.

Let us consider that a point in the region belongs to the route if it is within the error of the gravity acceleration, or three projections $\bar{g}$, or five independent components of the gravity tensor coincide.

We define the membership function of a point on the map grid for the designed route:

- for the acceleration amplitude:

(13) $$MF_{d,j}=MFM_{d,j}$$

- for the acceleration projection:

(14) $$MF_{d,j}=MFP_{x,d,j}\;\cdot \;MFP_{y,d,j}\;\cdot \;MFP_{z,d,j}$$

- for the tensor:

(15) $$MF_{d,j}=MFT_{xx,d,j}\;\cdot \;MFT_{yy,d,j}\;\cdot \;MFT_{xy,d,j}\;\cdot \;MFT_{xz,d,j}\;\cdot \;MFT_{yz,d,j}$$

If $MF_{d,j}$= 1, then the point is considered to conditionally belong to the route (i.e., used for further analysis), if $MF_{d,j}=0$, then the point is not used.

For the points selected by the criterion $MF_{d,j}$= 1, we also calculate the totals of the discrepancies of the vector projection:

(16) $$\Delta g_{d,j}=\Delta g_{x,d,j}+\Delta g_{y,d,j}+\Delta g_{z,d,j}$$

and of the tensor:

(17) $$\Delta T_{d,j}=\;\Delta T_{xx,d,j}+\;\Delta T_{yy,d,j}+\Delta T_{xy,d,j}+\Delta T_{xz,d,j}+\Delta T_{yz,d,j}$$

We enter the found points of the map, for which $MF_{d,j}$= 1, into a separate matrix, Res_MF, as shown in Table 7.

During the simulation, the Res_MF tables were calculated for different ε values over a wide alternating range: ±(0.01–100) mGal for the values $\left|\bar{g}\right|$, $g_{x}$, $g_{y}$, $g_{z}$ and ±(0.1–20) E for the values of the full gravity tensor components.

After determining the number $J_{d}$ of potential route points found and their positions relative to each other, the desired route among possible alternatives can be sought.

**Figure 4:**
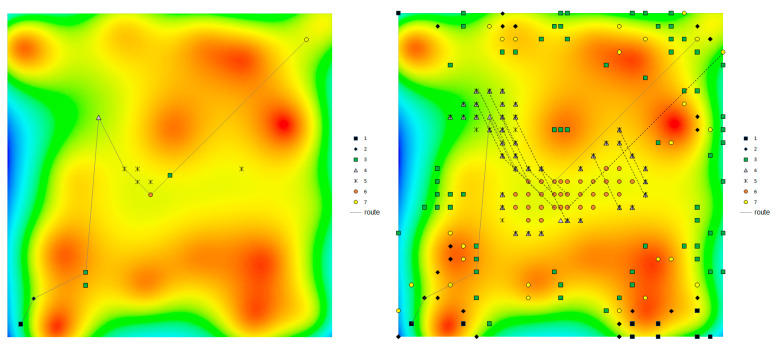

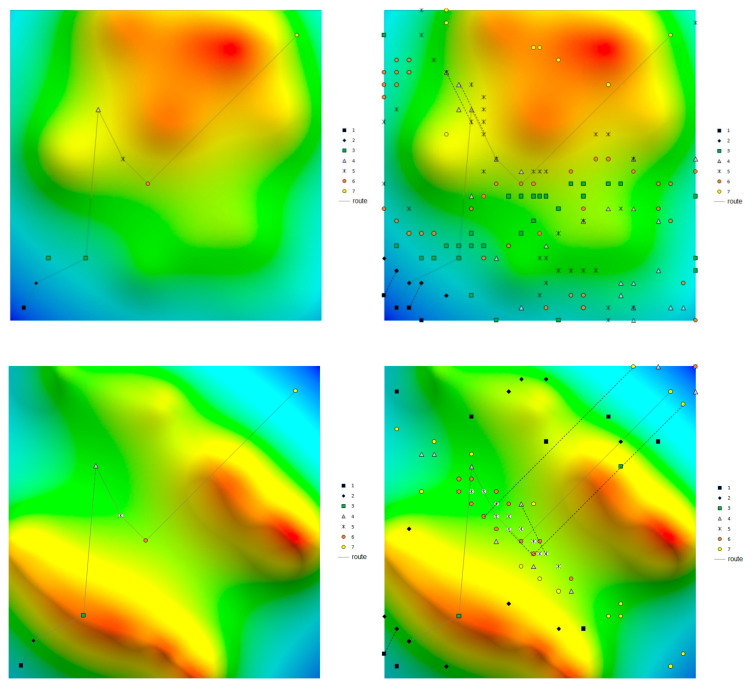
Potential route points found in the regions with low-, medium-, and high-amplitude anomalies with measurement errors of 0.2, 1, and 1 mGal, respectively, in the lines from top to bottom, respectively. The left column corresponds to the intersection of three projections of $\bar{g}$, while the right column corresponds to $\left|\bar{g}\right|$. The solid line denotes the true route. Points with the same d are denoted by the same markers.

The desired route is an aggregate of the found points with minimal differences in distances between adjacent points and the corresponding distances between points of the desired route (which can be considered to be obtained during movement between points). In this case, the route points should be as close as possible to the corresponding points of the reference region. If a route is found that is shifted in parallel by one or several steps of the grid relative to the real route, then it is an alternative route, as shown in Figure 4.

Thus, in the Res_MF matrix, we find such a set of points, D, the displacements between which $R_{MF,\;x,d+1,d}$ and $R_{MF,y,d+1,d}$ along the X and Y axes correspond, within an error δ, to the displacements $R_{x,d+1,d}$ and $R_{y,d+1,d}$ between the non referenced route.

In other words, we check the following:

(18) $$\begin{array}{c} R_{MF,\;x,d+1,d}-R_{x,d+1,d}\;\leq \;\delta \\ R_{MF,y,d+1,d}-R_{y,d+1,d}\;\leq \;\delta \end{array}$$

where

(19) $$\begin{array}{c} R_{x,d+1,d}=x_{d+1}-x_{d}\\ R_{y,d+1,d}=y_{d+1}-y_{d}\\ R_{MF,\;x,d+1,d}=x_{MF,d+1,j_{d+1}}-x_{MF,d,j_{d}}\\ R_{MF,y,d+1,d}=y_{MF,d+1,j_{d+1}}-y_{MF,d,j_{d}} \end{array}$$

Since in the simulation that we performed the route points corresponded to the grid points of the simulated region, δ = 0. The assumption of a zero error in movement detection between route points in our model is based on two factors. Firstly, 8 km grid spacing is too large compared to the real error in such measurements. Secondly, we wanted to focus on the analysis of gravity navigation errors associated with errors during the measurement of different characteristics of the gravity field.

An indicator of the effectiveness of the navigation algorithm is the number of routes built on the basis of points found in a given region for a given gravity characteristic.

If the search is conducted at the intersection of sets of the found points simultaneously for several characteristics (for example, for the projections $\bar{g}$ or tensor components), the points will be absent if for at least one of the characteristics, no values were found (based on the corresponding membership functions).

However, a more common case is the correspondence of several points at once to a single range of error. The more evenly the gravity characteristics are distributed, the more points and local regions of their distribution on the map will be found (Figure 4).

## 4. Simulation Results and Discussion

With a measurement error specified, the number of map points found by the criterion $MF_{d,j}=1$ for a given route point depends on both the overall distribution of anomalies on the map and on the anomaly amplitude at this point. However, the type of gravitational data used has the greatest influence on the number of points found.

### 4.1. Searching a Route by $\left|\bar{g}\right|$

Thus, when searching a route by $\left|\bar{g}\right|$, all route points were correctly found if the standard deviation of the virtual instrument did not exceed 0.1 mGal.

An increased standard deviation ε will result in new points with close values of $\left|\bar{g}\right|$. Since the field $\left|\bar{g}\right|$ does not have discontinuities, the found points will be grouped first around the true point of the route and, with a further increase in error, in other regions of the map with similar values of the field, as shown in Figure 4. Therefore, when searching a route according to the data from $\left|\bar{g}\right|$, regardless of the increase in the standard deviation of the virtual instrument, there are a priori correct points amongthe found points.

We denote $N_{true}$, $N_{\mathrm{false}}$,and $N_{potential}$ as the numbers of true, false, and potential points of the route found by the algorithm, corresponding to one point of the route, where $N_{potential}=\;N_{true}+N_{\mathrm{false}}$. In general, $N_{true}$ can be 0 or 1, and $N_{potential}$ can be 0 or any natural number.

**Figure 5:**
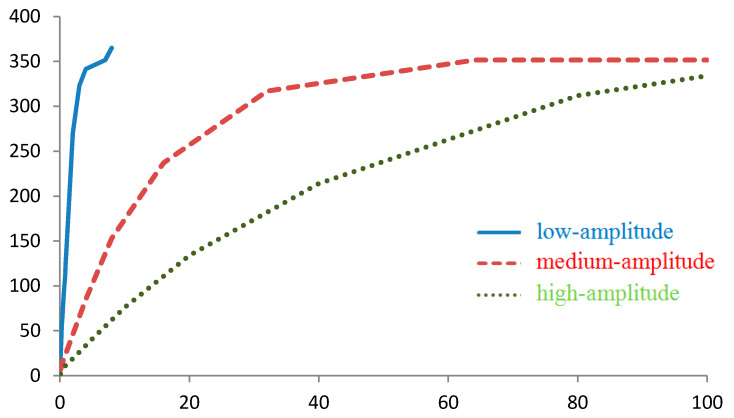
Dependence of the number of found potential points on the $N_{potential}$ route on the measurement error (mGal) for one route point when navigating by $\left|\bar{g}\right|$ for the case of regions with low-, medium-, and high-amplitude gravity anomalies.

The number $N_{potential}$, when navigating by $\left|\bar{g}\right|$, is large enough. Thus, in areas with weak, medium, and strong gravitational anomalies, a linear increase in the $N_{potential}\left(\varepsilon \right)$ dependence was observed with standard deviations of the virtual instrument up to 3, 8, and 20 mGal, respectively (Figure 5). At the same time, the slopes of $N_{potential}\left(\varepsilon \right)$ on the linear section were approximately 100, 20, and 10 points/mGal, respectively, for the indicated regions of anomalies, as shown in Figure 5.

For example, with a standard instrument deviation of 0.1 mGal, for each point of the route in areas with low-, medium-, and high-amplitude anomalies, $N_{potential}$ = 10, 2, and 1 points will be found on average, respectively.

Since, as indicated above, the correct points always belong to the set of found points, the route based on the data from $\left|\bar{g}\right|$ will be always found. However, with a relatively low value of ε, a cloud of false points will be found around each true point, which leads to the appearance of many alternatives to the true route, as shown in Figure 4. To distinguish the true route from the alternatives, additional comparisons of discrepancies by route points are required.

### 4.2. Searching a Route by $\bar{g}\left(g_{x},g_{y},g_{z}\right)$

If a route is searched by the condition of the intersection of three projections of the vector $\bar{g}\left(g_{x},\;g_{y},\;g_{z}\right)$, this situation will change. If a point is not found for at least one of the projections $\bar{g}$, then it will not be found at the whole intersection (according to the criterion $MF_{d,j}=1$). This condition will result not only in a decrease in the number of possible points of the route being found (approximately 10 times when compared with navigation by $\left|\bar{g}\right|$) but also to a decrease in the number of true route points found.

**Figure 6:**
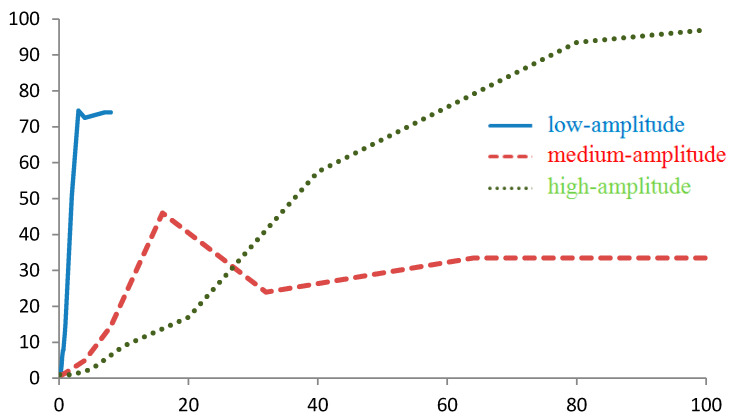
Dependence of the number of found potential points on the $N_{potential}$ route on the measurement error (mGal) for one route point when navigating by the intersection of three projections of the $\bar{g}$ vector for the case of regions with low-, medium-, and high-amplitude gravity anomalies.

The linearity of the $N_{potential}$(ε) dependence when navigating by three projections of $\bar{g}$ was observed to be in almost the same range as for navigation by $\left|\bar{g}\right|,$ up to the instrument standard deviation of 1, 4, and 20 mGal, for the regions of low-, medium-, and high-amplitude anomalies, respectively (Figure 6). However, the slopes of the $N_{potential}$(ε) curve were approximately 10, 2, and 1 points/mGal, respectively, i.e., 10 times less than when navigating by $\left|\bar{g}\right|$.

For example, when simulating navigation using data from the three projections of the vector $\bar{g}$, and with a virtual instrument standard deviation of 0.1 mGal, one route point contained on average approximately 1 to 2 potential route points in the regions with low-amplitude anomalies and approximately 1 potential point in the regions with medium- and high-amplitude anomalies.

**Figure 7:**
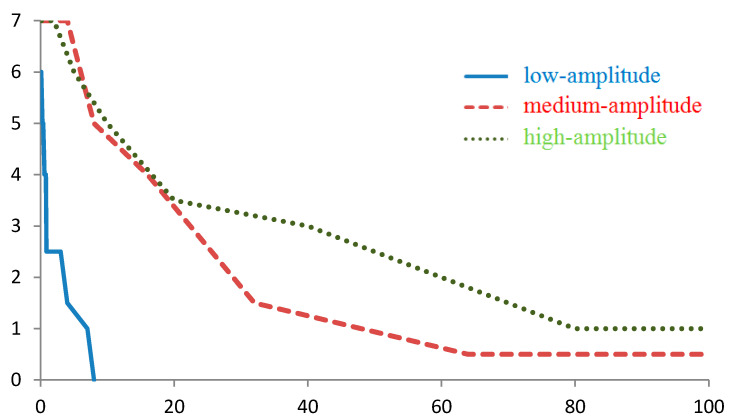
Dependence of the number $N_{true}$ of the correctly found route points on the measurement error (mGal) for a route containing 7 points when navigating by the intersection of the three projections of the $\bar{g}$ vector. Regions with low-, medium-, and high-amplitude gravity anomalies.

The number $N_{true}$ of the true route points decreased inversely with the standard deviation (Figure 7). In the linear approximation, the number of true points found for the entire route from 7 points decreases with increasing ε as 4, 0.5, and 0.25 points/mGal, for the low-, medium, and high-amplitude anomalies, respectively.

For the threshold criterion for the virtual device standard deviation, we can take its value at which at least half of the route points will be found. For the anomaly regions studied, the threshold values of ε were 1, 20, and 20 mGal, respectively.

### 4.3. Searching a Route by $T_{ij}$ ($T_{xx}$, $T_{xy}$, $T_{xz}$, $T_{yy}$, $T_{yz}$)

Navigation using the intersection of five independent tensor components sets an even more stringent criterion, that is, the desired route point must be found simultaneously for all five components. For this reason, with this type of navigation, false route points were generally absent in all simulated anomaly regions and for any standard deviation of the virtual instrument.

**Figure 8:**
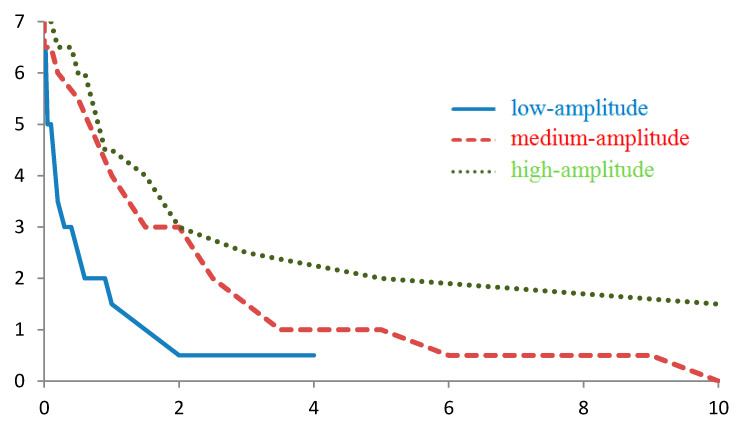
Dependence of the number $N_{true}$ of the correctly found route points on the measurement error (E) for a route containing 7 points when navigating using the intersection of five independent components of the gravity tensor. Regions with low-, medium-, and high-amplitude gravity anomalies.

The number of found true route points decreases when navigating by the projections g and is inversely proportional to the standard deviation of the virtual instrument. Using the linear approximation, for the entire route from 7 points, the average decrease in the number $N_{true}$ with increasing ε is 12, 3, and 2 points/E for regions with low-, medium, and high-amplitude anomalies, respectively (Figure 8).

Such a sharp decrease in the number of the true route points found with an increase in the standard deviation suggests that navigation based on the intersection of the tensor components places high demands on measurement errors since no algorithm that can find a route if its points are found.

When navigating using the tensor components, we take the value for which half of the true points of the route are lost for the limiting value of the standard deviation of the virtual instrument. For low-, medium-, and high-amplitude anomalies, these values were 0.2, 1, and 2 E, respectively (Figure 8).

### 4.4. Alternative Routes

**Figure 9:**
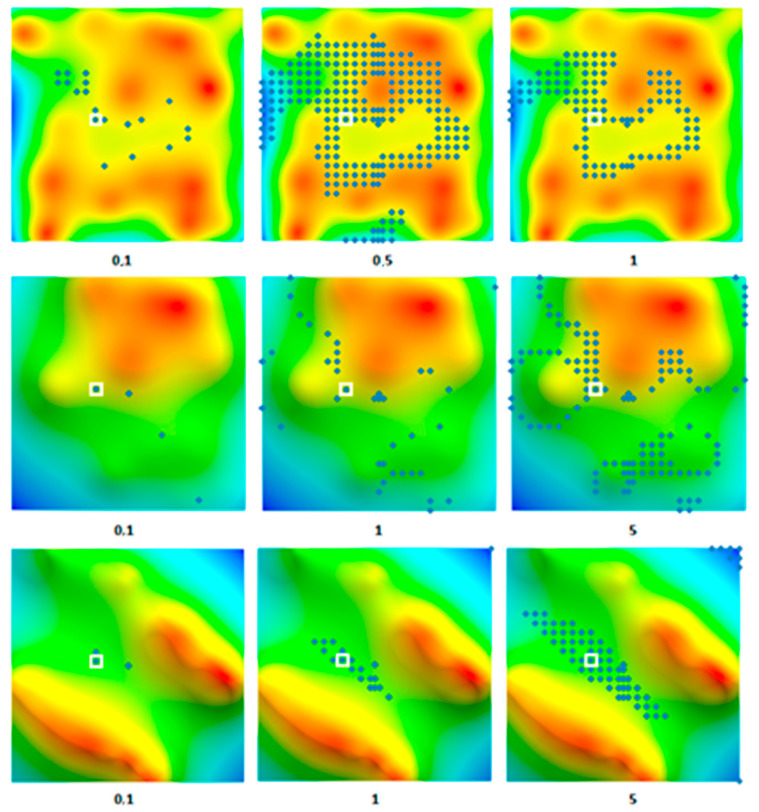
Distribution of the points found for the midpoint of the route by $\left|\bar{g}\right|$ distributions with different anomalies depending on the measurement error (indicated in mGal). The desired point is highlighted by the white square.

The number of alternative routes is directly related to the number of potential points found, which grows with an increase in the standard deviation of the virtual instrument and with a decrease in the magnitude (gradient) of the gravity anomalies, as shown in Figure 5, Figure 6 and Figure 9.

With the search algorithm based on $\left|\bar{g}\right|$, true points of the route are found for any measurement error, i.e., a threshold value of the standard deviation is absent, but for generality of reasoning, a standard deviation of the instrument of 1 mGal for this algorithm is used.

With the method of gravity navigation by $\left|\bar{g}\right|$ with a virtual instrument standard deviation of 1 mGal in regions with low-amplitude anomalies, no more than a two dozen independent alternative routes with lengths of 2 to 4 points were simultaneously found (Figure 4). These routes are immediately eliminated when the full-length (7 points) route is docked, i.e., they do not interfere with the determination of the true route. In regions with medium- and high-amplitude anomalies for this value of ε, alternative routes were completely absent.

When modeling the navigation using the three projections of the acceleration (with ε being equal to 1mGal), only a few alternative routes that were 2 points long were found in the low-amplitude anomaly region, and there were no alternative routes for regions with medium- and high-amplitude anomalies (Figure 4). These point-to-point routes can also be easily eliminated when building a full-length route.

For the method of gravity navigation by the five tensor components (with ε = 0.2 E), there were no alternative routes for all regions with different amplitudes of anomalies, because of the lack of false points of the route.

### 4.5. Features of Implementation of Gravity-Aided Navigation Based on Different Data Sources

(1) The method of navigating using the components of the field tensor is the most theoretically demanding in terms of hardware.

As has been mentioned above, in the regions with low-amplitude anomalies, this method will find half of the true points of the route if the standard deviation of the virtual instrument is not worse than 0.2 E (Figure 8). A gravity gradiometer, whose sensors are separated by a distance l=10 m, should have an accuratemeasurement of accelerations of at least 2 × 10^−4^ mGal.

For temperature drift on the order of 10 μg/°C [12] at a specified value of ε = 2 × 10^−4^ mGal, the arms of the gradiometer should be heat-stabilized to better than 2 × 10^−5^ °C, and the time drift and noise of the equipment should be maintained at this level. In addition, the accuracy of stabilization of the spatial position of the device must be at least 4”.

It is established by our simulation, with medium- and high-amplitude anomalies, the requirements for accuracy can be reduced by a factor of 5 to 6, but the hardware requirements remain notably high.

(2) When navigating using the three projections of vector g, the requirements for the permissible measurement error are considerably lower. Therefore, for smooth gravity fields, half of the true points of the route will be found with an error of 1 mGal with a grid spacing of 8 km (Figure 7). If the permissible navigation error is assumed to be 100 m, then the measurement errors of the g components should be approximately 0.01 mGal. Requirements for the temperature stability of the sensors are on the order of $10^{-3}$ °C. Spatial stabilization of the instrument is also needed, but its accuracy can be reduced to 30”.

Since for the regions with low-amplitude gravity anomalies the number of $N_{potential}$ points found for a single route point according to the data from the three projections of $\bar{g}$ increases with an increase in the standard deviation of the virtual instrument as 10 points/mGal with a grid spacing 8 km (Figure 6), then with a grid spacing 100 m and accuracy of 0.01 mGal, the number of points found will be approximately 10 × 80^2^ × 0.01 = 640.

If, however, the requirement for navigation accuracy is reduced to 1 km, then a measurement accuracy of 0.1 mGal is sufficient; the temperature stabilization requirement is 10^−2^ °C, and the spatial stabilization requirement is 100”, while for one point of the route, there will be approximately 10 × 8^2^ × 0.01 ≈ 10 points.

(3) The method of navigating by the value of $\left|\bar{g}\right|$ has the simplest hardware implementation since spatial stabilization of the position of the sensors is not required. Regardless of the position of the three orthogonal sensors, the vector $\bar{g}$ magnitude $\left|\bar{g}\right|$ can always be determined by measuringthe three projections. Only the requirements for temperature stabilization of the accelerometer sensors, as in the three projection method $\bar{g}$ (for example, 10^−2^ °C with a standard deviation of 0.1 mGal and navigation accuracy of 1 km), remain unchanged.

The issue with this method is the extremely high number of possible points found (Figure 5). Since the number of points found on an 8-km grid increases with an increase in the error in regions of low-amplitude anomalies as 100 points/mGal, then with an error of 0.1 mGal, 100 × 8^2^ × 0.1 = 640 points for one route point will be found on a 1-km grid. On a grid of 100 m with an error that is 10 times smaller, 10 times as many points will be found.

## 5. Conclusions

The simulations conducted show that global navigation based on gravimetric data is possible even when the largely imperfect route searching algorithm described in this report is applied. The choice of the data source is determined by both the measurement conditions and the accuracy of the positioning requirements. Nevertheless, the main strengths and weaknesses of each of the sources considered can be identified.

Thus, navigation by use of the full gravity tensor can provide the best positioning accuracy (up to tens of meters) with a minimal number of alternative solutions compared to navigation by the gravity acceleration magnitude or by the three orthogonal acceleration gravity projections. At the same time, navigation by tensor imposes the greatest demands on the measuring unit (the measurement accuracy should be sufficient to calculate the gravity gradient) and requires highly accurate spatial stabilization of the measuring equipment, which impedes the development and large-scale production of such systems.

Navigation based on the orthogonal projections of gravity imposes fewer requirements on the accuracy of the source data, while also requiring spatial stabilization. However, lower positioning accuracy with a significantly larger number of alternative found route points makes this method ineffective compared to tensor navigation.

Navigation based on the gravity acceleration magnitude with an efficiency at the level of navigation based on orthogonal projections does not require spatial stabilization and, in theory, makes it possible to rely on a considerably more compact hardware implementation than for the case of systems based on the gravity tensor. In theory, this approach makes it possible to consider this type of navigation as an auxiliary mechanism for unmanned aerial vehicles and other systems that require periodic validation for the operation of standard global navigation equipment.

## Figures and Tables

**Table 1:** Prototypes of the sites for building models and the corresponding distributions of the gravity potential. 1—LON [73–77.5]–LAT [64.5–66.5]. Western Siberia, Russia. 2—LON [70–74.5]–LAT [64–66]. Western Siberia, Russia. 3—LON [58–61]–LAT [48.3–50.3]. Mugodzhary Mountains with the Alabasskaya Basin, Kazakhstan.

No.	Prototype (Distribution $g_{z}$, mGal) [10]	Model (Distribution $g_{z}$, mGal)	Model (Gravity Potential, m^2^/s^2^)
1	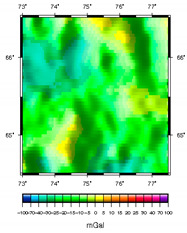	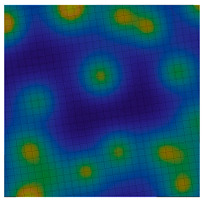	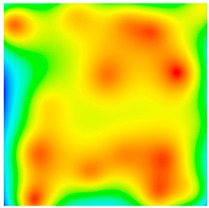
2	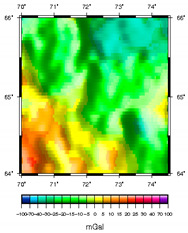	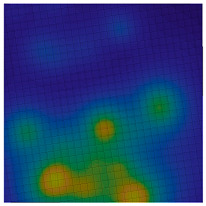	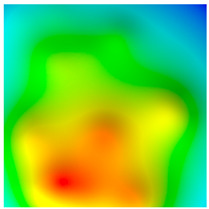
3	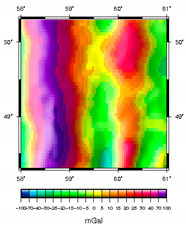	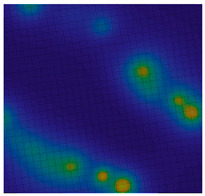	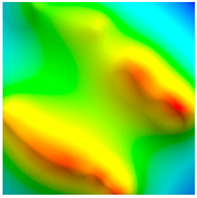

**Table 2:** True coordinates of the desired route points.

d	X, m	Y, m	Z, m
0	−92,000	−92,000	0
1	−84,000	−76,000	0
2	−52,000	−60,000	0
3	−44,000	36,000	0
4	−28,000	4000	0
5	−12,000	−12,000	0
6	84,000	84,000	0

**Table 3:** Route points characteristics for the region with low-amplitude anomalies.

Point Number	Accelerations, mGal				Tensor Components, E				
d	|g|	$g_{x}$	$g_{y}$	$g_{z}$	$T_{xx}$	$T_{yy}$	$T_{xy}$	$T_{xz}$	$T_{yz}$
0	10.6	7.0	0.8	−7.9	0.10	1.76	0.74	3.74	−1.02
1	8.9	4.6	−0.5	−7.6	1.88	−0.96	0.56	1.61	−1.22
2	6.4	−0.9	0.5	−6.3	−0.05	0.32	0.19	−1.25	0.22
3	3.7	0.8	−0.2	−3.6	−0.06	−0.46	−0.05	−0.02	−0.14
4	3.7	0.1	0.2	−3.7	−0.47	0.00	−0.44	−0.26	0.17
5	3.2	0.1	0.1	−3.1	−0.14	−0.51	−0.07	−0.03	0.11
6	7.8	0.1	0.4	−7.8	0.48	0.96	−1.49	1.65	1.70

**Table 4:** Route points characteristics for the region with medium-amplitude anomalies.

Point Number	Accelerations, mGal				Tensor Components, E				
d	|g|	$g_{x}$	$g_{y}$	$g_{z}$	$T_{xx}$	$T_{yy}$	$T_{xy}$	$T_{xz}$	$T_{yz}$
0	25.8	15.8	19.3	−6.7	−0.19	−1.65	−2.10	1.02	1.62
1	33.2	19.4	24.3	−11.8	0.56	−2.20	−2.43	1.82	3.67
2	41.0	20.6	27.0	−23.0	−3.30	0.37	2.60	2.09	3.00
3	54.8	23.1	−6.4	−49.3	−1.37	−2.91	−7.97	0.80	−5.80
4	56.5	7.1	25.9	−49.7	−9.00	5.08	−6.50	−2.10	6.90
5	47.4	7.3	28.8	−37.0	0.51	−6.95	0.00	0.90	6.45
6	83.5	−64.0	−24.3	−47.8	−8.71	9.37	−2.97	−20.59	−3.01

**Table 5:** Route points characteristics for the region with high-amplitude anomalies.

Point Number	Accelerations, mGal				Tensor Components, E				
d	|g|	$g_{x}$	$g_{y}$	$g_{z}$	$T_{xx}$	$T_{yy}$	$T_{xy}$	$T_{xz}$	$T_{yz}$
0	94.4	57.1	65.4	−37.2	−2.21	−5.53	−10.46	9.25	10.73
1	135.8	77.3	82.6	−75.2	−1.63	−3.58	−11.40	22.38	23.84
2	207.1	18.2	5.5	−206.2	17.10	63.87	36.74	0.63	−18.28
3	48.2	24.3	2.6	−41.5	0.83	−9.35	−4.96	3.45	6.46
4	40.4	9.2	−14.2	−36.7	−4.24	−7.31	−7.10	0.44	−0.72
5	41.5	3.4	−12.9	−39.3	−4.84	−8.12	−9.81	0.12	−1.74
6	56.2	−36.5	−40.4	−14.1	−1.40	−4.07	−4.64	−2.55	−2.74

**Table 6:** Matrix of the non-referenced route.

Rout Point Number	Route Point Coordinates Not Referenced to the Map			Values of the Gravity Field Characteristics Measured at the Route Points								
d	$x_{d}$	$y_{d}$	$z_{d}$	${\left|\bar{g}\right|}_{d}$	$g_{x,d}$	$g_{y,d}$	$g_{z,d}$	$T_{xx,\;d}$	$T_{yy,\;d}$	$T_{xy,\;d}$	$T_{xz,\;d}$	$T_{yz,\;d}$
0	$x_{0}$	$y_{0}$	0	${\left|\bar{g}\right|}_{0}$	$g_{x,0}$	$g_{y,0}$	$g_{z,0}$	$T_{xx,\;0}$	$T_{yy,\;0}$	$T_{xy,\;0}$	$T_{xz,\;0}$	$T_{yz,\;0}$
1	$x_{1}$	$y_{1}$	0	${\left|\bar{g}\right|}_{1}$	$g_{x,1}$	$g_{y,1}$	$g_{z,1}$	$T_{xx,\;1}$	$T_{yy,\;1}$	$T_{xy,\;1}$	$T_{xz,\;1}$	$T_{yz,\;1}$
*…*	*…*	*…*	*…*	*…*	*…*	*…*	*…*	*…*	*…*	*…*	*…*	*…*
D	$x_{D}$	$y_{D}$	0	${\left|\bar{g}\right|}_{D}$	$g_{x,D}$	$g_{y,D}$	$g_{z,D}$	$T_{xx,\;D}$	$T_{yy,\;D}$	$T_{xy,\;D}$	$T_{xz,\;D}$	$T_{yz,\;D}$

**Table 7:** Point searching results.

Route Point Number	Sequential Number of the Point Found on the Map, Corresponding to the Route Point d by the Criterion $MF_{d,j}=1$	Coordinates of the Points Found by the Criterion $MF_{d,j}=1$ for Route Point d, $j_{d}$ in Succession for Point d			Acceleration Magnitude Discrepancies, or Total of Vector Projection Discrepancies, or Total of Tensor Mismatch Discrepancies for the Route Point
d	$j_{d}$	$x_{MF,d,j_{d}}$	$y_{MF,d,j_{d}}$	$z_{MF,d,j_{d}}$	$\Delta {\left|\bar{g}\right|}_{d,j_{d}}$ or $\Delta g_{d,j_{d}}$ or $\Delta T_{d,j_{d}}$
0	1	$x_{MF,0,j_{1}}$	$y_{MF,0,j_{1}}$	0	$\Delta {\left|\bar{g}\right|}_{0,j_{1}}$ or $\Delta g_{0,j_{1}}$ or $\Delta T_{0,j_{1}}$
0	2	$x_{MF,0,j_{2}}$	$y_{MF,0,j_{2}}$	0	$\Delta {\left|\bar{g}\right|}_{0,j_{2}}$ or $\Delta g_{0,j_{2}}$ or $\Delta T_{0,j_{2}}$
…	…	…	…	…	…
0	$J_{0}$	$x_{MF,0,J_{0}}$	$y_{MF,0,J_{0}}$	0	$\Delta {\left|\bar{g}\right|}_{0,J_{0}}$ or $\Delta g_{0,J_{0}}$ or $\Delta T_{0,J_{0}}$
1	1	$x_{MF,1,j_{1}}$	$y_{MF,1,j_{1}}$	0	$\Delta {\left|\bar{g}\right|}_{1,j_{1}}$ or $\Delta g_{1,j_{1}}$ or $\Delta T_{1,j_{1}}$
1	2	$x_{MF,1,j_{2}}$	$y_{MF,1,j_{2}}$	0	$\Delta {\left|\bar{g}\right|}_{1,j_{2}}$ or $\Delta g_{1,j_{2}}$ or $\Delta T_{1,j_{2}}$
…	…	…	…	…	…
1	$J_{1}$	$x_{MF,1,J_{1}}$	$y_{MF,1,J_{1}}$	0	$\Delta {\left|\bar{g}\right|}_{1,J_{1}}$ or $\Delta g_{1,J_{1}}$ or $\Delta T_{1,J_{1}}$
…	…	…	…	…	…
D	$J_{D}$	$x_{MF,D,J_{D}}$	$y_{MF,D,J_{D}}$	0	$\Delta {\left|\bar{g}\right|}_{D,J_{D}}$ or $\Delta g_{D,J_{D}}$ or $\Delta T_{D,J_{D}}$
